# A High-Sensitivity Troponin I Rapid Assay vs. a High-Sensitivity Troponin T Routine Assay in Acute Chest Pain Patients: A Prospective Monocentric Study †

**DOI:** 10.3390/jcm14103456

**Published:** 2025-05-15

**Authors:** Emilie Han, Mariann Gyöngyösi, Elaaha Anwari, Vian Kokabi, Anna Gramser, Andreas Spannbauer, Monika Fritzer-Szekeres, Jutta Bergler-Klein

**Affiliations:** 1 Department of Cardiology, University Clinic of Internal Medicine 2, Medical University of Vienna, Währinger Gürtel 18–20, 1090 Vienna, Austria; 2 Department of Transfusion Medicine and Cell Therapy, Medical University of Vienna, Währinger Gürtel 18–20, 1090 Vienna, Austria; 3 Department of Laboratory Medicine, Medical University of Vienna, Währinger Gürtel 18–20, 1090 Vienna, Austria

**Keywords:** high-sensitivity troponin assay, troponin I, troponin T, acute myocardial infarction, cardiac enzymes

## Abstract

**Background/Objective**: The measurement of troponin is recommended for acute myocardial infarction (AMI) diagnosis. Yet, hs-cardiac troponin T (hs-cTnT) can be elevated due to non-cardiac conditions, such as skeletal muscle injury, chronic kidney disease (CKD) or pulmonary embolism. The aim of our study was to compare the diagnostic accuracy of a bedside rapid hs-cardiac troponin I (hs-cTnI) assay (Quidel TriageTrue^®^) with hs-cTnT measured in a routine laboratory (Roche Elecsys). **Methods**: This prospective monocentric study was conducted in an acute cardiac outpatient unit at a tertiary hospital. Hs-cTnI was measured via a point-of-care test from whole blood, while hs-cTnT was measured from plasma through the routine laboratory facility. **Results**: In 129 patients (65.1% male, 61.8 ± 15.6 years) with acute chest pain, results for hs-cTnI were available 14 ± 11 min after the first clinical presentation, which was 74 ± 54 min earlier than for hs-cTnT. Coronary angiography confirmed AMI in 17 patients (13.28%). The relative risk of AMI patients with elevated hs-cTnI results was 6.59 compared to 2.29 for hs-cTnT. Hs-cTnI exhibited an equivalent negative predictive value to hs-cTnT (99%) for AMI but had a comparatively higher positive predictive value (50.0 vs. 25.8%). In 39 patients with at least CKD stage 3a, median hs-cTnT was pathological (27.0 ng/L), in contrast with hs-cTnI (11.2 ng/L). Further, hs-cTnI was less likely elevated in patients with CKD and no AMI. **Conclusions**: The diagnostic value of hs-cTnI was comparable to that of hs-cTnT, and the blood sampling-to-result time was shorter than routine hs-cTnT.

## 1. Introduction

Cardiac troponins (cTns) are proteins expressed in cardiac muscle cells which enable the interaction between actin and myosin and are therefore fundamental to muscle contraction. The protein complex consists of cardiac troponin I (cTnI), cardiac troponin C (cTnC) and cardiac troponin T (cTnT). Cardiac troponin levels in the blood are extremely low in healthy subjects which is explained by the low turnover of cardiac muscle cells [1]. In the case of myocardial injury, elevated cTn levels can be measured in blood and is associated with a worse adverse prognosis in both cardiac and non-cardiac diseases [2,3].

The 2018 Fourth Universal Definition of Myocardial Infarction states that myocardial injury is detected when at least one value above the 99th percentile upper reference limit (URL) is measured in a patient with high-sensitivity methods for cTnI or cTnT [4,5]. Although elevated cTn values reflect injury to myocardial cells, they do not indicate the underlying pathophysiological mechanisms, but various causes have been suggested for the release of structural proteins from the myocardium [6,7]. Yet, it is not clinically possible to distinguish which increases in cTn levels are due to which mechanisms in the acute or chronic setting [8]. Recently, high-sensitivity assays for the rapid quantification of cTnI and cTnT from serum, plasma and whole blood have been developed for rapid near patient testing in the emergency setting. Point-of-care tests of troponin in the emergency setting may have the added benefit of reducing the time to diagnosis and the treatment of myocardial infarction (MI) even further [9,10,11].

The comparability of high-sensitivity cardiac troponin assays is influenced by specimen type (whole blood vs. plasma), which cardiac troponin targets to measure (cTnI versus cTnT), and manufacturer-specific 99th percentile thresholds, which may be subject to age, gender and ethnicity [12,13]. Thus, it is necessary to evaluate the prognostic value of different high-sensitivity point-of-care tests for the quantification of cTn in varying clinical settings and populations [14,15]. The aim of this study was to compare the diagnostic accuracy of a rapid assay for high-sensitivity quantification of hs-cTnI using a whole blood specimen versus central laboratory testing of hs-cTnT.

## 2. Materials and Methods

### 2.1. Patient Population and Study Setting

This article is a revised and expanded version of a conference paper entitled “Comparison of rapid test for high-sensitivity troponin I with laboratory high-sensitivity troponin T in patients with acute chest pain—a prospective monocentric study”, which was presented at the ESC Congress London between 30 August 2024 and 2 September 2024 [16]. This prospective monocentric study was conducted in the acute cardiac outpatient unit of the Department of Cardiology at the General Hospital of Vienna, a tertiary hospital, where patients with emergent cardiac symptoms were evaluated for diagnosis and treatment. This study was performed in accordance with the Declaration of Helsinki and received approval from the Ethics Committee of the Medical University of Vienna (EC reference number: 2352/2019, approval date: 26 March 2021). Informed consent was obtained from all included patients. Inclusion criteria consisted of patients who were at least 18 years old with suspected myocardial infarction or myocardial injury requiring a troponin test, informed about the aims and scope of the study by an authorized physician and signed the informed consent form. Exclusion criteria comprised pregnancy or nursing women and the refusal to participate. A total of 149 consecutive patients were prospectively included between May 2021 and August 2022. Of those, 20 subjects were later excluded in the statistical evaluation due to withdrawal of consent (*n* = 6) or invalid troponin test results (*n* = 14); thus, results are presented from the data of 129 patients (more details in Supplementary Figure S1).

### 2.2. Specimen Collection and Testing

The first 175 µL of the blood sample was used for the immediate testing of hs-cTnI in the acute cardiac outpatient unit using the Quidel TriageTrue^®^ High Sensitivity Troponin I Test (Quidel Germany GmbH, Kornwestheim, Germany), followed by plasma sampling for the testing of hs-cTnT at the clinical central laboratory (Roche Elecsys, Rotkreuz, Switzerland). The high-sensitivity assay for the quantification of cTnI suitable for decentralized testing of plasma, serum or whole blood specimens had a reportable range of 1–1000 ng/L and a 99th percentile URL of 20.5 ng/L overall, with values of 14.4 ng/L for women and 27.5 ng/L for men, respectively [17]. For measurements of whole blood using this hs-cTnI assay, the limit of detection was 1.5–1.9 ng/L, while the limit of quantitation (20% CV) was 2.80 ng/L, and the concentration range at 10% CV was 5.8–6.2 ng/L. Testing in the acute cardiac outpatient unit was conducted by nurses, medical students and physicians. For hs-cTnT, the 99th percentile URL of the laboratory method is 14 ng/L for both male and female patients. The hs-cTnT assay had a limit of detection of 2.97 ng/L, a limit of quantitation (20% CV) of 2.30 ng/L, a concentration at 10% CV of 5.40 ng/L and a reportable range of 3 ng/L to 10,000 ng/L [18].

### 2.3. Additional Clinical and Laboratory Parameters

The following parameters were recorded for all patients: age, body height, body weight, comorbidities, heart rate, systolic and diastolic blood pressure, N-terminal pro–B-type natriuretic peptide (NT-pro-BNP), creatinine, hemoglobin, C-reactive protein, d-Dimer, fibrinogen, creatine kinase and hematocrit. The body mass index (BMI) was calculated for each patient as kg/m^2^. Echocardiography and other clinical diagnostic procedures were conducted depending on the clinical case and clinical necessity (see Section 2.4). Documented echocardiographic parameters comprised the presence of left ventricular hypertrophy, aortic valve stenosis, aortic regurgitation, mitral valve stenosis, mitral regurgitation and tricuspid regurgitation (ranked by severity grade: 0 = absent, 1 = mild, 1.5 = mild to moderate, 2 = moderate, 2.5 = moderate to severe and 3 = severe). The glomerular filtration rate (GFR) was calculated using the CKD-EPI equation. Chronic kidney disease (CKD) was considered present if patients had known CKD stage 3a or higher [19].

### 2.4. Clinical Diagnosis

Patients with suspected MI underwent routine diagnostic and therapeutic work-up according to the current guidelines at that time [20]. Clinical examination, patient history and 12-lead electrocardiogram (ECG) were assessed for all patients at presentation. Depending on the clinical case and medical need, further investigations included echocardiography, coronary angiography with or without intervention, computerized tomography, chest X-ray and cardiac magnetic resonance tomography for a final diagnosis. In cases of ST-segment elevation myocardial infarction (STEMI), no serial troponin measurements were acquired as they went directly to the heart catheter lab. In patients presenting with elevated troponin levels and persistent symptoms, continued observation with repeat ECGs and serial troponin measurements was carried out to detect dynamic ischemic changes using the 0/3 h hs-TnT algorithm as part of the routine work-up. The definite diagnosis of type 1 MI was confirmed by the presence of observable plaques with a stenosis of 50% or more in coronary angiography. If an alternative diagnosis (i.e., pulmonary embolism, cardiac decompensation, panic attack, etc.) was established for the cause of chest pain, patients were treated accordingly or transferred. The decision to discharge patients with elevated troponin levels but without type 1 myocardial infarction was made on an individual basis by the treating clinician, in line with current guidelines. This decision was supported by symptom resolution, the absence of new or evolving ECG changes and a stable troponin trend. A medical chart review at least a month after the acute cardiac outpatient unit visit was performed to arrive at the final diagnosis of MI or no MI. The time between symptom onset and the first blood draw for troponin testing was not recorded.

### 2.5. Statistical Analyses

Categorical variables were presented as counts and percentages, while continuous variables were reported as the mean ± standard deviation or median with a range from the minimum to the maximum according to their distribution.

For statistical evaluation, hs-cTnI and hs-cTnT were stratified into two groups: “normal” and “elevated”. The cut points for hs-cTnI were gender-specific 99th percentile URL values of 14.4 ng/L for women and 27.5 ng/L for men. For the laboratory hs-cTnT test, the 99th percentile URL of 14 ng/L was applied as the cut point for both male and female patients. Agreement was assessed between hs-cTnI and hs-cTnT using contingency tables, Spearman’s correlation and the Bland–Altman plot (with upper and lower limits of agreement). Furthermore, accuracy, positive predictive and negative predictive values were reported for hs-cTnI and hs-cTnT to be able to rule-in MI.

Additionally, the patients were stratified into five groups depending on the presence of MI and corresponding hs-cTnI and hs-cTnT levels: patients with diagnosed MI (group 1, *n* = 17); No MI, with hs-cTnI and hs-cTnT both elevated (group 2, *n* = 14); No MI, with hs-cTnI normal and hs-cTnT elevated (group 3, *n* = 31); No MI, with hs-cTnI elevated and hs-cTnT normal (group 4, *n* = 2); and No MI, with hs-cTnI and hs-cTnT both normal (group 5, *n* = 65).

Comparisons of hs-cTnI and hs-cTnT with selected comorbidities between patients with MI (group 1) and without MI (groups 2–4) were performed using the Kruskal–Wallis test and the Wilcoxon test. Statistical analyses were performed using R, version 4.0.5 (R Foundation for Statistical Computing, Vienna, Austria), and Prism (Version 10.2.0). A two-tailed *p* value of <0.05 was considered significant.

## 3. Results

### 3.1. Patient Characteristics

Of the 129 subjects eligible for statistical evaluation, 84 (65.12%) were male and 45 (34.88%) were female. Seventeen (13.28%) patients were diagnosed with MI; of those, eleven had STEMI, while six had non-ST-segment elevation myocardial infarction (NSTEMI). Five (29.4%) MI patients were female, while the mean age was 60.5 ± 13.6 years and comparable overall. Patients with MI displayed variable extents of stenotic events (1–3 vessel diseases) in the left anterior descending coronary artery (LAD), the proximal right coronary artery (RCA) and the left circumflex coronary artery (LCX). Hypertrophy, aortic valve stenosis, aortic regurgitation, mitral stenosis, mitral regurgitation and tricuspid regurgitation were frequently observed to co-occur in the group of patients with MI.

Overall, the measurement of hs-cTnI using a rapid test directly in the acute cardiac outpatient unit yielded quick results by 14 ± 11 min after blood sampling and was 74 ± 54 min faster compared to routine hs-cTnT measurements. Measurements of hs-cTnT (14 ng/L) showed a higher median value compared to hs-cTnI (7 ng/L) (Table 1). The median value of both hs-cTnT and hs-cTnI for patients with MI was above the URL (median for hs-cTnT: 891 ng/L and median for hs-cTnI: 1000 ng/L).

### 3.2. Agreement Between Laboratory Hs-cTnT and Bedside Hs-cTnI

Agreement rates in terms of elevated and not elevated results between the test for hs-cTnT and hs-cTnI are shown in Tables S1 and S2. The overall agreement rate between hs-cTnI and hs-cTnT for results above the 99th percentile URL of the related test was 46.77% (95% CI: 34.16–59.79) and 95.52% (95% CI: 86.63–98.83) for results below the 99th percentile URL. An elevated result for hs-cTnI in the acute cardiac outpatient unit using the point-of-care test predicted a positive hs-cTnT test result in the routine laboratory in 90.63% of patients.

**Figure 1:**
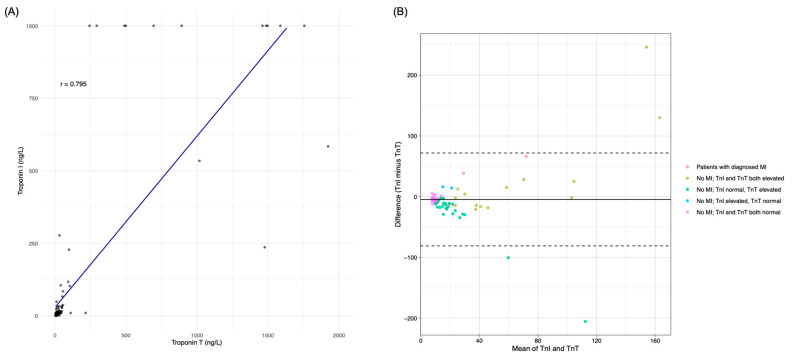
(**A**) Correlation plot between troponin I and troponin T, with a correlation coefficient of r = 0.795 and y = 29.31 + 0.59x; (**B**) Bland–Altman plot of high-sensitivity cardiac troponin I (hs-cTnI) versus high-sensitivity cardiac troponin T (hs-cTnT) measurements. Mean values of troponin I and troponin T are plotted against the difference between measurements (hs-cTnI minus hs-cTnT). The upper and lower limits of agreement (LOA) are represented by the dotted horizontal lines; within those lines, 95% limits of agreement are represented (mean bias ±1.96 × SD of mean difference). Average difference (solid line): −4.6 ng/L (95% CI, −12.4 to 3.2); upper LOA: 71.8 ng/L (95% CI, 58.3 to 85.3); lower LOA: −81.1 ng/L (95% CI, −94.6 to −67.5). The values outside of the reliably quantifiable areas (hs-cTnT < 4 ng/L, hs-cTnI < 0.1 ng/L and hs-cTnI > 1000 ng/L) were excluded from the Bland–Altman plot and analysis. The colors of the points represent the five different groups of patients with and without myocardial infarction (MI) and their respective hs-cTnI and hs-cTnT measurements: (i, red dots) patients with diagnosed MI; (ii, olive dots) No MI, with hs-cTnI and hs-cTnT both elevated; (iii, green dots) No MI, with hs-cTnI normal and hs-cTnT elevated; (iv, blue dots) No MI, with hs-cTnI elevated and hs-cTnT normal; and (v, pink dots) No MI, with hs-cTnI and hs-cTnT both normal.

Hs-cTnI and hs-cTnT had a Spearman correlation coefficient of r = 0.795 (Figure 1A). The Bland–Altman plot displays very good agreement between hs-cTnI and hs-cTnT for all patients with a definite MI diagnosis (Figure 1B). The outliers above and below the upper and lower limits of agreement (LOA) in the Bland–Altman plot are patients from group 2 (No MI, with hs-cTnI and hs-cTnT both elevated) and group 3 (No MI, with hs-cTnI normal and hs-cTnT elevated), and these patients had several comorbidities, such as CKD, atrial fibrillation or a previous mitral valve replacement. The average difference between hs-cTnI and hs-cTnT measurements was −4.6 ng/L (95% CI: −12.4 to 3.2), while the upper and lower LOA were 71.8 ng/L (95% CI: 58.3 to 85.3) and −81.1 ng/L (95% CI: −94.6 to −67.5), respectively.

### 3.3. Comparison of Hs-cTnT and Hs-cTnI for Differentiation of Patients with and Without MI

Both tests displayed elevated hs-cTn values in 16 of 17 patients diagnosed with MI, resulting in a sensitivity of 94.12% (95% CI: 69.23–99.69) (Table 2 and Table 3). One male patient with an anteroseptal myocardial infarction did not display elevated hs-cTnT (10 ng/L) above the 99th percentile URL of the assay (14 ng/L), whereas the hs-cTnI test conducted in the acute cardiac outpatient unit was elevated with a value of 48 ng/L (a 99th percentile URL of 27.5 ng/L). The second patient diagnosed with an acute subendocardial myocardial infarction displayed a slight elevation in the hs-cTnT test (26 ng/L), while hs-cTnI did not show an elevation (12 ng/L).

The specificity of the hs-cTnI assay was significantly higher compared to the hs-cTnT assay, with 85.71% (95% CI: 77.54–91.26) for hs-cTnI and 58.93% (95% CI: 49.23–68.01) for hs-cTnT, which can be appreciated in a better positive predictive value (PPV) for hs-cTnI than hs-cTnT. The negative predictive values (NPVs) were equivalent for both tests, with values of 98.97 and 98.51 for hs-cTnI and hs-cTnT, respectively.

**Figure 2:**
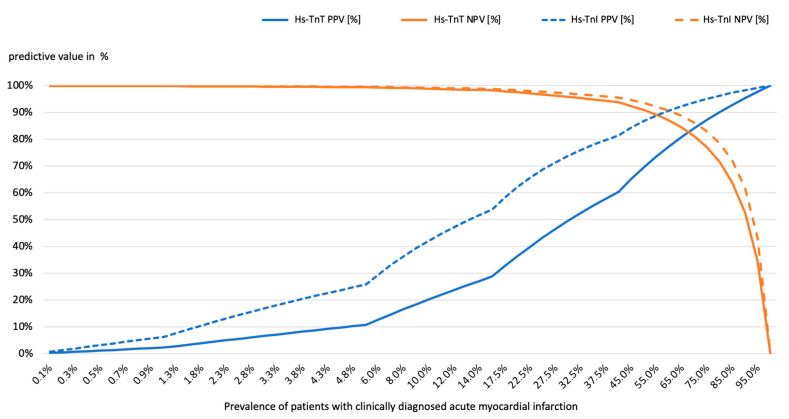
Positive and negative predictive values of hs-cTnI and hs-cTnI to rule-in or rule-out myocardial infarction (MI) while respecting a prevalence range of 0.1% to 100.0% diagnosed MI. Solid lines represent hs-TnT, while dashed lines represent hs-TnI. The PPV is shown in blue, and the NPV is shown in orange. TnI—troponin I, TnT—troponin T, NPV—negative predictive value and PPV—positive predictive value.

As a result, the relative risk of patients with diagnosed MI versus without diagnosed MI to receive an elevated hs-cTnI result was 6.59 for the hs-cTnI test compared to 2.29 for the hs-cTnT-test, meaning that a positive hs-cTnI test result was a better classifier for true MI than the hs-cTnT test. In a calculated prevalence range of 0.1–100% of diagnosed myocardial infarctions, a PPV of an elevated hs-cTnI test result that would correctly rule-in an MI would be higher than that of an elevated hs-cTnT test result, while the NPV would be equivalent for both tests (Figure 2).

### 3.4. Comparison of Hs-cTnT and HSs-cTnI in Terms of Findings from Clinical Diagnostics

Echocardiography findings included the assessment of left ventricular (LV) hypertrophy, aortic valve stenosis, aortic valve calcification, aortic regurgitation, mitral stenosis, mitral regurgitation and tricuspid regurgitation (mainly physiological or minimal). LV hypertrophy was detected in 54 patients, of which twenty had mild, five mild to moderate, seventeen moderate, one moderate to severe and eleven severe assessments of myocardial thickening. Aortic valve stenosis, aortic valve calcification and aortic regurgitation were summarized as aortic valve disease, while mitral valve stenosis and mitral regurgitation were summarized as mitral valve disease (Table 4).

**Figure 3:**
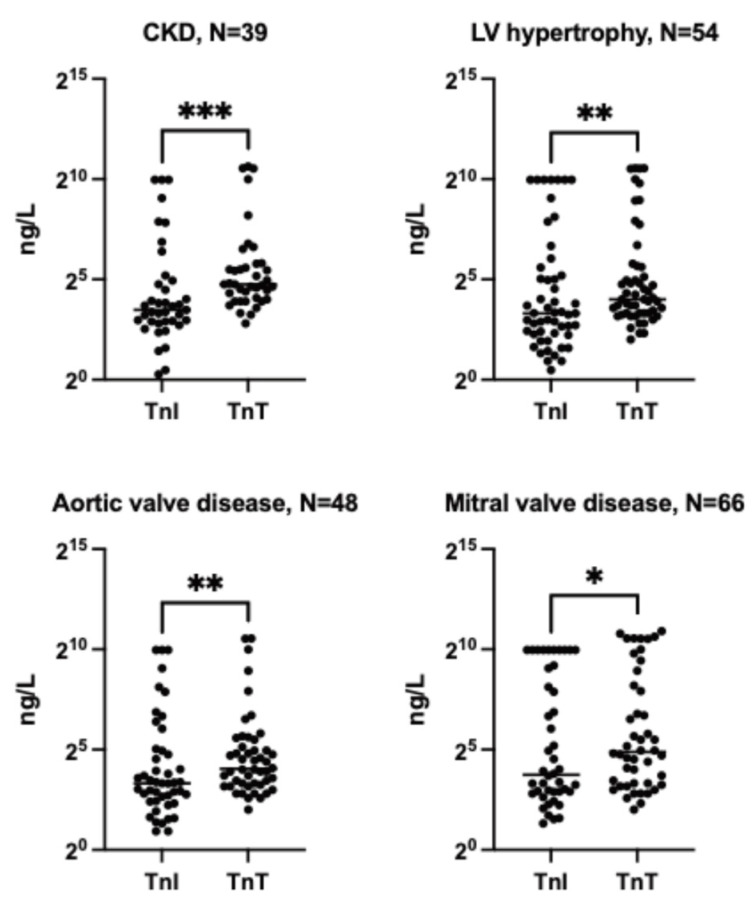
Comparison of hs-cTnI and hs-cTnT in patients with other clinical conditions. Troponin I values were comparably lower than troponin T values in the presence of CKD, LV hypertrophy, aortic valve disease and mitral valve disease. CKD—chronic kidney disease (of at least grade 3a) and LV—left ventricular. Wilcoxon test: * *p* ≤ 0.05, ** *p* ≤ 0.01 and *** *p* ≤ 0.001.

**Figure 4:**
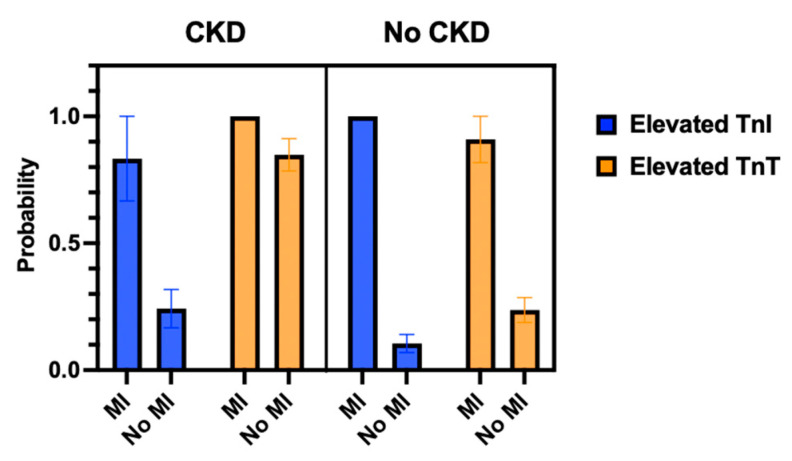
Patients with chronic kidney disease without MI have a lower probability of elevated hs-cTnI compared to elevated hs-cTnT. Mean probability of elevated troponin I or troponin T values in CKD patients with and without MI. Mean and SEM as error bars. CKD—chronic kidney disease and MI—myocardial infarction.

Median hs-cTnI measurements were lower compared to hs-cTnT measurements in patients with CKD, LV hypertrophy, aortic valve disease and mitral valve disease (Figure 3). Furthermore, hs-cTnT was more likely to be elevated in patients with CKD without MI compared to hs-cTnI (Figure 4).

## 4. Discussion

This study provides real-world data and investigates the use of the Quidel TriageTrue^®^ High Sensitivity Troponin I test in an acute cardiac outpatient unit using the whole blood of patients with acute chest pain while comparing assay performance and turnaround time with the central laboratory hs-cTnT test. Our study revealed three main findings: (1) the accuracy of the hs-cTnI assay (0.87) to detect true MI was higher compared to hs-cTnT (0.64) in our patient population; (2) the test results for hs-cTnI were available 74 min faster on average compared to hs-cTnT; (3) in CKD patients, hs-cTnT is elevated even without MI, whereas hs-cTnI better differentiates MI from non-MI cases.

Hs-cTnI was shown to possess better diagnostic performance for MI in early presentations (<3 h since pain onset) and higher accuracy to predict a positive angiographic finding; however, hs-TnT performed better in later presentations and was a more accurate predictor for mortality [21]. Nevertheless, Klinkenberg et al. investigated the diurnal rhythm of cTn and its consequences for the diagnosis of acute myocardial infarction. As a result, hs-cTnT measurements, but not hs-cTnI, exhibited a diurnal rhythm, characterized by gradually decreasing concentrations throughout daytime, rising concentrations during night times and peak concentrations in the morning (mean 16.2 ng/L at 8:30 AM and 12.1 ng/L at 7:30 PM). The authors concluded that even if the diurnal hs-cTnT rhythm does not affect the diagnostic accuracy of hs-cTnT for acute myocardial infarction, it should be considered when using hs-cTnT for screening purposes [22]. In our study, when MI prevalence was 13.28%, the NPVs of hs-cTnI and hs-cTnT were nearly identical, with 98.97% (95% CI: 93.57–99.95) for hs-cTnI and 98.51% (95% CI: 90.08–99.92) for hs-cTnT. Thus, both tests are equally reliable in ruling out MI, regardless of disease prevalence given the reported sensitivity and specificity values. However, unlike hs-cTnT, hs-cTnI is less influenced by circadian fluctuations, making it a more stable marker for consistent assessments.

Our findings on the diagnostic accuracy of the hs-cTnI assay in an independent cohort of 129 chest pain patients are in line with the recently published findings from the Mersey Acute Coronary Syndrome Rule-Out Study (MACROS-2), which evaluated the TriageTrue^®^ High Sensitivity Troponin I assay in a larger population of 1157 patients in the UK [23]. MACROS-2 reported a sensitivity of 95.7%, a specificity of 93.6%, a PPV of 58.4%, and an NPV of 99.0% for the diagnosis of acute myocardial infarction (AMI), compared to a sensitivity of 94.12%, a specificity of 85.71%, a PPV of 50.00%, and an NPV of 98.97% in our study. The slight differences in specificity and PPV between the two studies may be attributed to variations in patient populations and study settings. In MACROS-2, the median age was 59 years, and 47.2% were female, and 15% had a history of previous MI. In contrast, our study population had a median age of 63 years, with 34.9% female participants and 22.5% with a known history of coronary artery disease. These demographic differences could influence the pre-test probability of AMI and, consequently, the diagnostic performance metrics. Moreover, Dakshi et al. demonstrated that whole-blood hs-cTnI measurements achieved high diagnostic accuracy, with AUC values ranging from 0.95 to 0.99 at different time points, comparable to both a central laboratory hs-cTnI assay (Siemens Atellica) and an established hs-cTnT assay (Roche Elecsys) [23]. The lack of standardization between hs-cTn point-of-care assays limits direct comparability [24], but the demonstrated reliability of hs-cTnI POC testing across clinics and patient groups herein supports its role in ruling out early MI and workflow improvement.

Furthermore, a recent study showed that a test for the quantification of hs-cTnT (Roche Elecsys) showed significant higher values for TnT in patients with skeletal muscle disorders (SMDs) versus control subjects without SMDs (median: 16 ng/L with an interquartile range (IQR) of 7–32.5 ng/L versus 5 ng/L (an IQR of 3–9 ng/L; *p* < 0.001)), whereas the complementary assay for hs-cTnI did not show significant higher values. hs-cTnT–Elecsys concentrations were above the upper limit of normal in 55% of patients with SMDs versus 13% of control subjects (*p* < 0.01) [2]. Biochemical data indicate that injured skeletal muscle expresses proteins that are detected by the hs-cTnT assay, leading to some situations where elevations of hs-cTnT could originate from skeletal muscle [25,26,27]. Recent data suggest that the frequency of such elevations in the absence of ischemic heart disease may be higher than originally thought [28,29].

Additionally, hs-cTnI may be better suitable for ruling in MI in patients with known CKD, as patients with CKD stage 3a or higher had elevated hs-cTnT in a non-MI setting more frequently than elevated hs-cTnI. Higher cut-off levels for patients with CKD have been proposed for this reason, which have shown improved accuracy in the diagnosis of NSTEMI in severe CKD patients [30]. Moreover, the guidelines of the Fourth Universal Definition of Myocardial Infarction propose to use an increase in cTn of greater than 20% in CKD patients with troponin levels greater than the 99th percentile of the URL as rule-in for MI [4].

A recent study investigated the incidence and prognostic relevance of periprocedural myocardial injury (PMI) in patients with NSTEMI after percutaneous coronary intervention (PCI) using cTnI as a diagnostic biomarker. Periprocedural ischemic events were frequent in patients with NSTEMI undergoing PCI (37.4%), with prognostic implications indicating worse survival (adj. hazard ratio: 2.68) and a higher incidence of major adverse cardiac events (MACEs) (adj. hazard ratio: 1.39) [31]. A post-PCI of ΔcTnI >40%, combined with an absolute postprocedural value of ≥5 times the 99th percentile upper reference limit, was identified as the optimal threshold for diagnosing prognostically relevant PMI using chemiluminescent immunoassay for cTnI (Beckman Coulter Access AccuTnI + 3 assay) and hs-cTnI (Access hsTnI assay, Beckman Coulter) [31]. The potential use of a POC hs-cTnI assay for the early detection of PMI in this context is an important area for future research, offering a practical approach for improving post-procedural risk stratification and outcomes.

To conclude, a rapid whole-blood hs-cTnI test showed comparable accuracy to laboratory hs-cTnT for detecting myocardial infarction, with a higher positive predictive value and an equivalent negative predictive value, supporting its use for early diagnosis in emergency settings. Nonetheless, further multicenter cohort studies with a broader patient population and settings with procedural variations compared to ours are required to provide further evidence for the findings presented in this study.

### Limitations

Limitations include the single-center aspect and the retrospective nature of this study without formal power analysis, which may recruit a biased patient population. Further, the number of true MIs in this reported patient cohort of acute chest pain symptoms was low. This could be due to the fact that the study was conducted when measures to contain COVID-19 including social distancing were in place at that time in Austria, which may have influenced patients’ willingness to visit the hospital despite symptoms and rather visiting primary physicians instead. Thus, due to the small sample size of the study population, the potential generalizability to a wider population could be decreased. Further, the time between symptom onset and blood draw can significantly impact measured troponin concentrations, which we did not record systemically for our analyses. Several studies have investigated the reliability of patient-reported symptom onset times in AMI, particularly in STEMI. While patient reports are generally useful for initial clinical decisions, research indicates that they may not always precisely reflect the actual onset of ischemia, with an underestimation of ischemic time by patient-reported onset time occurring more often in high-risk patients [32].

## Figures and Tables

**Table 1:** Patient characteristics.

Characteristic	Patients with Diagnosed MI (*n* = 17)	Patients with No MI (*n* = 112)	Overall (*n* = 129)
**Female**	5 (29.4%)	40 (35.7%)	45 (34.9%)
**Age**	60.5 (13.6)	61.9 (15.9)	61.8 (15.6)
**BMI**	27.2 (3.43)	28.3 (6.11)	28.1 (5.71)
Missing	1 (5.9%)	41 (36.6%)	42 (32.6%)
**hs-cTnI** in ng/L, Median [Min, Max]	1000 [12.9, 1000]	6.05 [0.1, 277]	7.00 [0.100, 1000]
**hs-cTnT** in ng/L, Median [Min, Max]	891 [10.0, 1920]	12.0 [4.0, 215]	14.0 [4.00, 1920]
**Coronary artery disease (known)**	7 (41.2%)	22 (19.6%)	29 (22.5%)
Missing	0 (0%)	2 (1.8%)	2 (1.6%)
**NTproBNP** in pg/mL, Median [Min, Max]	812 [49.5, 14,400]	302 [5.0, 13,100]	342 [5.00, 14,400]
Missing	2 (11.8%)	1 (0.9%)	3 (2.3%)
**GFR (CKD-EPI)** in ml/min/1.73 m^2^, Median [Min, Max]	80.6 [14.0, 111]	77.5 [8.39, 129]	77.7 [8.39, 129]
Missing	0 (0%)	3 (2.7%)	3 (2.3%)
**Hemoglobin** in g/dL	12.9 (2.04)	13.7 (1.8)	13.6 (1.82)
Missing	0 (0%)	3 (2.7%)	3 (2.3%)
**Hematocrit** in %	38.6 (5.35)	41.0 (5.2)	40.6 (5.28)
Missing	0 (0%)	3 (2.7%)	3 (2.3%)
**Creatine kinase** in U/L, Median [Min, Max]	267 [36.0, 1320]	88.5 [12.0, 2920]	99.0 [12.0, 2920]
Missing	1 (5.9%)	4 (3.6%)	5 (3.9%)
**C-reactive protein** in mg/dL, Median [Min, Max]	0.30 [0.04, 7.56]	0.15 [0.03, 26.10]	0.165 [0.03, 26.10]
Missing	0 (0%)	3 (2.7%)	3 (2.3%)
**Fibrinogen** in mg/dL	388 (119)	365 (90.4)	368 (94.5)
Missing	1 (5.9%)	7 (6.3%)	8 (6.2%)
**Systolic blood pressure** in mmHg	125 (22.6)	136 (26.5)	135 (26.3)
Missing	12 (70.6%)	11 (9.8%)	23 (17.8%)
**Diastolic blood pressure** in mmHg	75.4 (17.0)	83.5 (17.0)	83.2 (17.0)
Missing	12 (70.6%)	11 (9.8%)	23 (17.8%)
**Heart rate** in beats per minute (bpm)	66.8 (13.6)	82.3 (20.6)	81.0 (20.5)
Missing	9 (52.9%)	25 (22.3%)	34 (26.4%)

Descriptive statistics are shown as counts and percentages (%) and mean (SD) if not specified otherwise. MI—myocardial infarction, GFR—glomerular filtration rate and SD—standard deviation.

**Table 2:** Accuracy and predictive values of the hs-cTnT test for the inclusion and exclusion of myocardial infarction (MI).

		MI		Total
		yes	no	
**Troponin T**	**Elevated**	16	46	62
	**Not elevated**	1	66	67
**total**		17	112	129

**Parameter**		**%**	**95% CI**	
Sensitivity for true MI		94.12	69.24–99.69	
Specificity for exclusion of MI		58.93	49.23–68.01	
Positive predictive value		25.81	15.89–38.73	
Negative predictive value		98.51	90.08–99.92	
Risk for elevated hs-cTnT with MI		0.94		
Risk for elevated hs-cTnT without MI		0.41		
Relative risk for elevated hs-cTnT (MI vs. non-MI)		2.29		

**Table 3:** Accuracy and predictive values of the hs-cTnI test for the inclusion and exclusion of myocardial infarction (MI).

		MI		Total
		yes	no	
**Troponin I**	**Elevated**	16	16	32
	**Not elevated**	1	96	97
**total**		17	112	129

**Parameter**		**%**	**95% CI**	
Sensitivity for true MI		94.12	69.23–99.69	
Specificity for exclusion of MI		85.71	77.54–91.36	
Positive predictive value		50.00	32.24–67.76	
Negative predictive value		98.97	93.57–99.95	
Risk for elevated hs-cTnI with MI		0.94		
Risk for elevated hs-cTnI without MI		0.14		
Relative risk for elevated hs-cTnI (MI vs. non-MI)		6.59		

**Table 4:** Comorbidities stratified by patients with and without myocardial infarction (MI).

Comorbidity	Overall (*n* = 129)	MI (*n* = 17)	No MI (*n* = 112)	*p*-Value
**Chronic kidney disease**	39 (30.2%)	6 (35.3%)	33 (29.5%)	n.s.
**Left ventricular hypertrophy**	54 (48.2%)	11 (64.7%)	43 (38.4%)	0.040
**Aortic valve disease**	48 (37.2%)	6 (35.3%)	42 (37.5%)	n.s.
**Mitral valve disease**	66 (51.2%)	12 (70.6%)	54 (48.2%)	n.s.

MI—myocardial infarction.

## Data Availability

The original contributions presented in this study are included in the article and its supplementary material. Further inquiries can be directed to the corresponding author.

## Supplementary Materials

The following supporting information can be downloaded at: https://www.mdpi.com/article/10.3390/jcm14103456/s1, Figure S1. Study flowchart of the inclusion and exclusion of patients and blood testing for troponin T and troponin I; Table S1. Agreement between hs-cTnI and hs-cTnT (overall and stratified by male/female); Table S2. Percent agreement between hs-cTnI and hs-cTnT, predictive values and likelihood ratios (overall and stratified by male/female).

## Abbreviations

The following abbreviations are used in this manuscript:

AMI	Acute myocardial infarction
CKD	Chronic kidney disease
ECG	Electrocardiogram
GFR	Glomerular filtration rate
LCX	Left circumflex coronary artery
LAD	Left anterior descending artery
LOA	limits of agreement
LV	Left ventricular/ventricle
Hs-cTnI	Hs-cardiac troponin I
Hs-cTnT	Hs-cardiac troponin T
NPV	Negative predictive value
NSTEMI	Non-ST-segment elevation myocardial infarction
NTproBNP	N-terminal pro–B-type natriuretic peptide
PCI	Percutaneous coronary intervention
PMI	Periprocedural myocardial injury
PPV	Positive predictive value
RCA	Right coronary artery
RR	Relative risk
SMDs	Skeletal muscle disorders
STEMI	ST-segment elevation myocardial infarction
URL	Upper reference limit
