# Supplementary material for: A High-Sensitivity Troponin I Rapid Assay vs. a High-Sensitivity Troponin T Routine Assay in Acute Chest Pain Patients: A Prospective Monocentric Study
Source: J Clin Med. 2025 May 15;14(10):3456. doi: 10.3390/jcm14103456 (PMC12112038; doi:10.3390/jcm14103456)
Supplement: Supplementary file 1 [file jcm-14-03456-s001.zip › jcm-3598394-supplementary.pdf]

Supplementary Materials

Article title

A High-Sensitivity Troponin I Rapid Assay vs. a High-Sensitivity Troponin T Routine Assay in Acute Chest Pain Patients: A Prospective Monocentric Study †

Supplementary Figure S1. Study flowchart of the inclusion and exclusion of patients and blood testing for troponin T and troponin I.

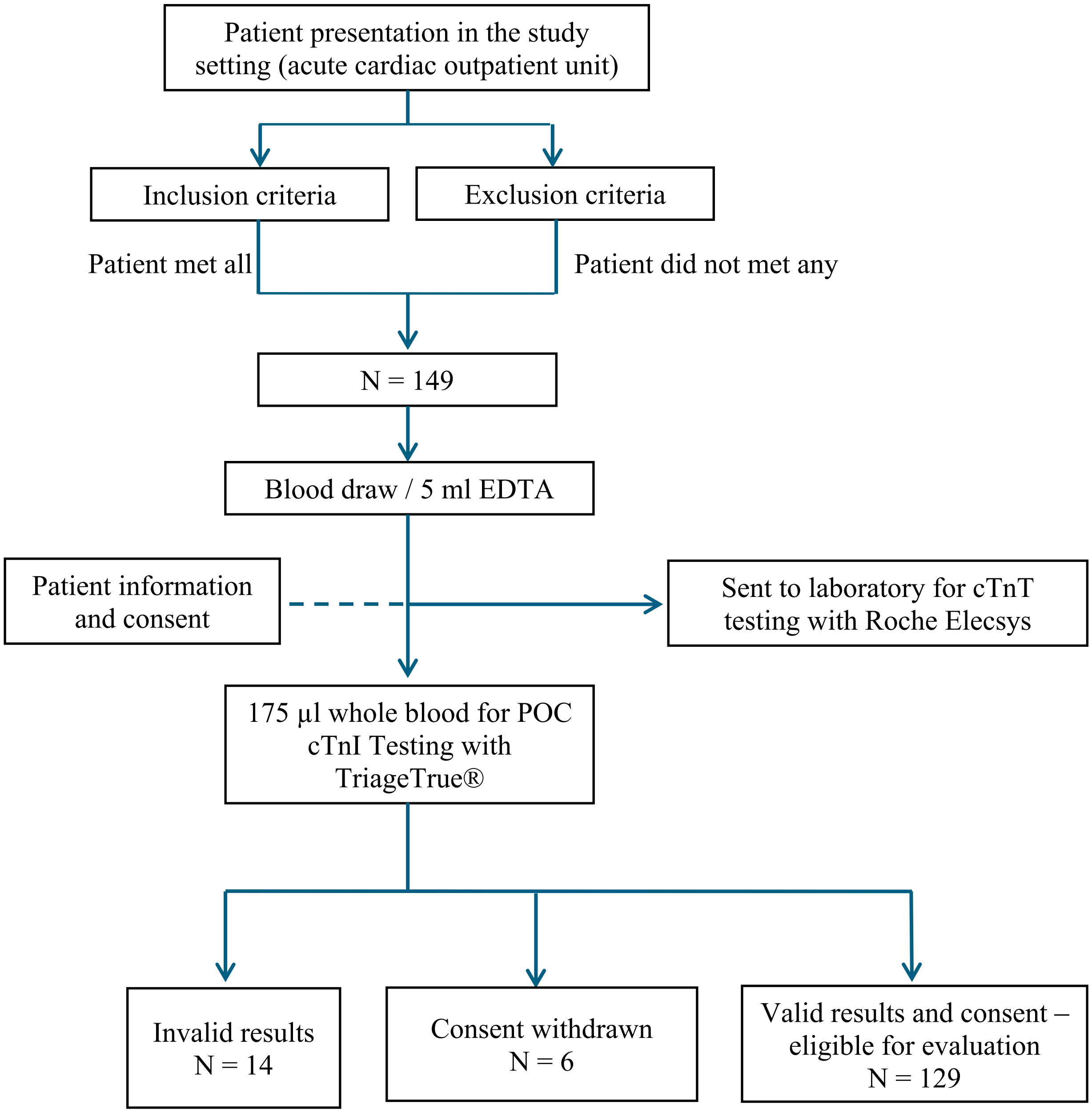

Supplementary Table S1. Agreement between hs-cTnI and hs-cTnT (overall and stratified by male/female).

Comparison of hs-cTnT measured in lab versus hs-cTnI measured bedside		hs-cTnI					
		Male		Female		Overall	
		elevated	normal	elevated	normal	elevated	normal
hs-cTnT	elevated	21	22	8	11	29	33
	normal	2	39	1	25	3	64
	total	23	61	9	36	32	97
Prevalence of elevated hs-cTnT (%)		51.19		42.22		48.06	

Supplementary Table S2. Percent agreement between hs-cTnI and hs-cTnT, predictive values and likelihood ratios (overall and stratified by male/female).

Table 3	Male		Female		Overall	
	%	95% CI	%	95% CI	%	95% CI
**Agreement of elevated results between hs-cTnI and hs-cTnT**	48.84	33.56 - 64.32	42.11	21.12 - 66.03	46.77	34.16 - 59.79
**Agreement of non-elevated results between hs-cTnI and hs-cTnT**	95.12	82.19 - 99.15	96.15	78.42 - 99.79	95.52	86.63 - 98.83
**PPV of hs-cTnI**	91.30	70.49 - 98.48	88.89	50.67 - 99.42	90.63	73.83 - 97.55
**NPV of hs-cTnI**	63.93	50.57 - 75.54	69.44	51.72 - 83.08	65.98	55.58 - 75.10
**Positive likelihood ratio (weighted by prevalence)**	10.50	2.78 - 39.71	8.00	1.24 - 51.51	9.67	3.27 - 28.55
**Negative likelihood ratio (weighted by prevalence)**	0.56	0.39 - 0.80	0.44	0.26 - 0.74	0.52	0.39 - 0.69

cTnI – cardiac troponin I, cTnT – cardiac troponin T, hs – high-sensitivity, NPV – negative predictive value, PPV – positive predictive value.
